# *TbMYC4A* Is a Candidate Gene Controlling the Blue Aleurone Trait in a Wheat-*Triticum boeoticum* Substitution Line

**DOI:** 10.3389/fpls.2021.762265

**Published:** 2021-11-05

**Authors:** Xin Liu, Minghu Zhang, Xiaomei Jiang, Hui Li, Zhenjiao Jia, Ming Hao, Bo Jiang, Lin Huang, Shunzong Ning, Zhongwei Yuan, Xuejiao Chen, Xue Chen, Dengcai Liu, Baolong Liu, Lianquan Zhang

**Affiliations:** ^1^Triticeae Research Institute, Sichuan Agricultural University, Chengdu, China; ^2^State Key Laboratory of Crop Gene Exploration and Utilization in Southwest China, Sichuan Agricultural University, Chengdu, China; ^3^Qinghai Province Key Laboratory of Crop Molecular Breeding, Xining, China; ^4^Key Laboratory of Adaptation and Evolution of Plateau Biota (AEPB), Northwest Institute of Plateau Biology, Chinese Academy of Sciences, Xining, China

**Keywords:** *Triticum boeoticum*, anthocyanin biosynthesis, blue aleurone, transcriptome analysis, *TbMYC4A*

## Abstract

*Triticum boeoticum* Boiss (A^b^A^b^, 2n = 2x = 14) is one of the sources of the blue grain trait controlled by *blue aleurone layer 2* (*Ba2*). However, the underlying genes have not been cloned. In this study, a transcriptomic comparison between a blue-grained wheat-*T. boeoticum* substitution line and its wheat parent identified 41 unigenes related to anthocyanin biosynthesis and 29 unigenes related to transport. The bHLH transcription factor gene *TbMYC4A* showed a higher expression level in the blue-grained substitution line. *TbMYC4A* contained the three characteristic bHLH transcription factor domains (bHLH-MYC_N, HLH and ACT-like) and clustered with genes identified from other wheat lines with the blue grain trait derived from other Triticeae species. *TbMYC4A* overexpression confirmed that it was a functional bHLH transcription factor. The analysis of a *TbMYC4A-*specific marker showed that the gene was also present in *T. boeoticum* and *T. monococcum* with blue aleurone but absent in other Triticeae materials with white aleurone. These results indicate that *TbMYC4A* is a candidate gene of *Ba2* controlling the blue aleurone trait. The isolation of *TbMYC4A* is helpful for further clarifying the genetic mechanism of the blue aleurone trait and is of great significance for breeding blue-grained wheat varieties.

## Introduction

Colored-grain wheat is regarded as an ideal food for human health and has attracted increasing interest from both food manufacturers and researchers. Unlike purple-grained wheat, which stores anthocyanins in the seed coat, blue-grained wheat shows anthocyanin accumulation in the aleurone layer (Abdel-Aal and Hucl, 2003, Abdel-Aal et al., 2006). In addition, purple and blue wheat can be crossed to form black wheat (BW), which is rich in nutritional and bioactive compounds giving it great potential for the development of healthy functional foods (Dhua et al., 2021). Anthocyanins are an important component of the human diet and are widely distributed in vegetables and fruits. They plan an important role in human health as anti-inflammatory, antioxidant, anticancer and antidiabetic agents (Shipp and Abdel-Aal, 2010). Anthocyanidins require glycosylation, methylation, and acylation to form stable anthocyanins and over 400 anthocyanins have been described so far (Springob et al., 2003; Sasaki et al., 2013). There are six main anthocyanidins in plants: delphinidin, cyanidin, peonidin, pelargonidin, petunidin and malvidin (Kähkönen and Heinonen, 2003). Delphinidin is the major anthocyanidin in blue-grained wheat (Trojan et al., 2014).

Common wheat does not contain the blue grain trait, but the trait can be introduced by crossing it with some species of Triticeae to give it a blue aleurone layer (Burešová et al., 2015). Several genes conferring the blue aleurone trait have been transferred into wheat from homoeologous group 4 of Triticeae species (Burešová et al., 2015). *Ba1* is derived from *Thinopyrum ponticum*, located on the long arm of chromosome 4Ag FL 0.71–0.8 (Zheng et al., 2006; Liu et al., 2018). *ThMYC4E* is a candidate *Ba1* gene (Li et al., 2017). *Ba2*, originating from *Triticum boeoticum* and *Triticum monococcum*, was mapped to a location close to the centromere on the long arm of 4A^b^ or 4A^m^, respectively (Zeller et al., 1991; Zeven, 1991; Dubcovsky et al., 1996; Singh et al., 2007; Yu et al., 2017). *BaThb* is located on the chromosome 4J of *Th. bessarabicum* (Shen et al., 2013). In addition, the *HvMYC2* gene was identified to be involved in anthocyanin synthesis in the barley aleurone layer (Strygina et al., 2017), and *TsMYC2* has been correlated with the blue grain trait in triticale (Zong et al., 2019).

The anthocyanin biosynthesis pathway is generally an extension of the flavonoid pathway and is a component of secondary plant metabolism (Larter et al., 2019; Wheeler and Smith, 2019). In many plants, the anthocyanin biosynthesis pathway is a highly conserved network (Tanaka et al., 2008). Anthocyanin biosynthesis begins with the conversion of phenylalanine to coumaric CoA through a three-step enzymatic reaction. Then, the chalcone synthase (CHS)-mediated synthesis of naringin chalcone from malonyl-CoA and 4-coumaroyl-CoA occurs. Chalcone isomerase isomerizes (CHI) chalcone to naringenin. Naringin is hydroxylated to form dihydroflavanols, which is catalyzed by flavonoid-3-hydroxylase (F3H), and can be further hydroxylated by flavonoid 3′,5′-hydroxylase (F3′5′H) or 3′-hydroxylase (F3′H) to generate dihydromyricetin or dihydroquercetin, respectively. Colored anthocyanidins are formed by dihydroflavonol 4-reductase (DFR) and anthocyanin synthase (ANS/LDOX). Subsequently, various stable anthocyanins are formed by modification by acyltransferase (AT), methyltransferase (MT), and glucosyltransferase (GT) (Springob et al., 2003; Sasaki et al., 2013). After the synthesis of anthocyanins in the cytosol, they must enter the vacuole for their diverse colors to be observable (Winkel-Shirley, 2001). One of the most complete potential mechanisms of anthocyanin transport involves the cooperation of glutathione transferase (GST) and multidrug resistance-associated related proteins (MRPs) (Goodman et al., 2004; Hu et al., 2016). MYB, bHLH and WD40 transcription factors form the MBW complex and combine with promoters to activate the transcription of structural genes in the anthocyanin biosynthetic pathway, thereby driving the tissue-specific accumulation of pigments (Ravaglia et al., 2013; Lloyd et al., 2017). Studies have shown that the co-expression of MYB and bHLH transcription factors can induce the synthesis of anthocyanins, such as *ZmC1* and *ZmR* in maize and *TaMYB7D* and *TaMYC1* in purple wheat (Ahmed et al., 2003; Zong et al., 2017).

The key genes for anthocyanin biosynthesis have been isolated in distinct tissues of certain plants using high-throughput RNA sequencing (RNA-Seq) (Li et al., 2017; Strygina et al., 2017; Zong et al., 2019). In our previous study, the blue-grained wheat-*T. boeoticum* substitution line Z18-1244 was developed (Liu et al., 2020). However, the gene controlling the blue aleurone of *T. boeoticum* has not been identified. The objectives of the present study were (1) to compare the expression levels of transcripts in the aleurone of red- and blue-grained wheat and (2) to identify the candidate genes regulating the blue aleurone of the Z18-1244 substitution line.

## Materials and Methods

### Plant Materials

The wheat-*T. boeoticum* substitution line Z18-1244 and its parents (common wheat Crocus and *T. boeoticum* G52) were used for RNA-Seq analysis. A total of 24 genotypes were subjected to *TbMYC4A* gene detection, including 14 with white aleurone from *Triticum turgidum*, *Aegilops tauschii*, *Triticum urartu*, *Triticum araraticum*, and *Triticum zhukovskyi* and 10 with blue aleurone from *T. boeoticum* and its cultivated type, *T. monococcum* (Supplementary Table 1). Crocus and G52 were kindly provided by George Fedak at the Ottawa Research and Development Center in Canada. Lines with PI or CItr prefixes were kindly provided by USDA-ARS, United States, while AS lines were obtained from Sichuan Agricultural University. *Triticum zhukovskyi* TRI 7270-1 and TRI 7270-3 were kindly provided by Prof. Fangpu Han at the Institute of Genetics and Developmental Biology, Chinese Academy of Sciences.

### Genomic DNA, Total RNA, and cDNA Preparation

Genomic DNA was extracted from fresh leaves 22 days post-anthesis using a plant genomic DNA kit (Tiangen Biotech, Beijing Co. Ltd., Beijing, China). At 14, 22, 28, 35 days post-anthesis (dpa), the aleurone layer of the immature grains was carefully stripped and immediately placed in liquid nitrogen for total RNA extraction, with replicates of 10 grain aleurone layers for each material. Total RNA was extracted using the Tiangen RNAprep Pure Plant Kit (Tiangen Corporation, Beijing, China) according to the standard protocol. RNA concentration and purity were measured using a NanoDrop 2,000 spectrophotometer (Thermo Fisher Scientific, Wilmington, DE). RNA integrity was assessed using the RNA Nano 6000 Assay Kit of the Agilent Bioanalyzer 2100 system (Agilent Technologies, CA, United States). cDNA was obtained from total RNA using the Thermo RevertAid First Strand cDNA Synthesis Kit (Thermo-Fisher Scientific, Shanghai, China).

### Illumina Sequencing and Transcriptome Analysis

Total RNA at 22 days post-anthesis was purified and used to construct a Strand-Specific Normal Library for sequencing on the Illumina (HiSeq 2500/4000) platform (Biomarker Technologies Co., Ltd., China). The clustering of index-coded samples was performed on a cBot Cluster Generation System using the TruSeq PE Cluster Kit v4-cBot-HS (Illumina) according to the manufacturer’s instructions. After cluster generation, the library preparations were sequenced on an Illumina platform, and paired-end reads were generated.

Raw data (raw reads) in FASTQ format were first processed with in-house Perl scripts. All downstream analyses were based on clean data with high quality. These clean reads were then mapped to the reference genome sequence. Only reads with a perfect match or one mismatch were further analyzed and annotated based on the reference genome. HISAT2 tools were used for mapping against the reference genome (ta_IWGS C_MIPSv2.2_HighConf_CDS_2014Jul18.fa) (Kim et al., 2015). The average mapping ratio of each sample reached 86.27%, which showed that the data were comparable between samples. After comparative analysis, String Tie comparison was performed to assemble and quantify the reads (Pertea et al., 2015). Differential expression analysis was performed using the DESeq2 R package (Love et al., 2014). Differentially expressed genes (DEGs) were identified according to a false discovery rate (FDR) threshold of < 0.05 and a | log2 (fold change)| ≥ 1. To annotate and classify genes, the assembled unigenes were subjected to searches in public databases, including Nr,^1^ SWISS-PROT,^2^ COG,^3^ KEGG,^4^ GO,^5^ KOG,^6^ Pfam,^7^ and eggNOG.^8^ Gene ontology (GO) and KEGG enrichment analyses were performed using ShinyGO v 0.61 (Ge et al., 2020). The functional categories of the genes were selected according to an enrichment FDR of *p* < 0.05. Genes related to anthocyanin synthesis were collected from KEGG and compared with unigenes sequenced from blue and white aleurone by the BlastX algorithm with an *E*-value of < 1e-5. The raw sequence reads were stored in the NCBI SRA database with the accession number PRJNA757248.

### PCR Amplification

Primer 5 software (Premier Biosoft, Palo Alto, CA, United States) was used to design primers. PCR amplification was performed using Vereti96 Abi Life 2720 (Thermo Fisher Scientific) and i-Max Ultra-high-fidelity DNA polymerase (Hunan Innovagene Biotechnology Limited). The PCR reaction system and amplification program for all primers used in the manuscript are shown below: 2 μl DNA/cDNA (200 ng/μl), 1 μl F/R (10 mM), 1 μl i-Max,1 μl dNTPs (10 mM), 25 μl 2 × i-Max Buffer, add double distilled water to 50 μl and then denaturation at 95°C for 5 min, 35 cycles of 95°C for 30 s, 60°C for 30 s, an 72°C for 1 min and 40 s, followed by a final extension at 72°C for 5 min. The Tiangen TIAN gel Midi Purification Kit (Tiangen Co.) was used to purify the PCR products from 1% agarose gel. The PCR products were cloned into the pGEM-T Easy plasmid vector (Promega Corporation, Madison, WI, United States), and the recombinant plasmid was subsequently transformed into *Escherichia coli* DH5α cells, from which five positive clones were selected and sent to a commercial company (Tsingke Biotechnology Co., Ltd.) for sequencing. All primers used in this study are listed in Supplementary Table 2. The coding sequence of *TbMYC4A* was stored in National Center for Biotechnology Information^9^ under accession number MZ686958.

### Bioinformatics Analysis

The conserved functional domains were predicted on https://www.ncbi.nlm.nih.gov/Structure/cdd/wrpsb.cgi website. Phylogenetic trees of the amino acid sequences of bHLH transcription factors were constructed by using MEGA X via the adjacency matrix method (Kumar et al., 2018). The pairwise deletion parameters and the p-distance model were used. A bootstrap test of phylogeny was performed with 1,000 replicates.

### Transient Expression

Transient expression vectors were constructed using TbMYC4AF2 and TbMYC4AR2 as adapter-specific primers. The expression vector pLGY-02:TbMYC4A contained the whole coding sequence of *TbMYC4A* with a maize ubiquitin promoter. pBRACT214:TaMYC1 and pBRACT214:TaMYB7D, provided by the Northwest Institute of Plateau Biology, Chinese Academy of Sciences, have been shown to act together to induce the production of large numbers of red cells in the white wheat coleoptile (Zong et al., 2017). Following the steps of the gene gun bombardment technique described by Ahmed et al. (2003), different vector combinations were bombarded into the white coleoptile of Opata wheat with a gene gun, after which they were incubated in light for 16 h after bombardment, and all treated coleoptiles were observed through a stereomicroscope (Leica Co., Oskar Barnack, Germany), photographed and counted.

### Quantitative Real-Time PCR Analysis

*GAPDH* (Glyceraldehyde-3-phosphate dehydrogenase) was used as the reference to determine relative expression values (Scholtz and Visser, 2013), and quantitative real-time PCR (qPCR) was performed using the CFX96 PCR System. cDNA was synthesized from 1 mg of total RNA and diluted 50-fold in DEPC-treated water prior to qPCR analysis. The qPCR amplification was performed using TB Green^®^ Premix Ex Taq^TM^ II (TliRNaseH Plus), 10 μl of reaction mixture including 5 μl 2 × TB Green Premix Ex Taq II(Tli RNaseH Plus), 0.4 μl F/R (10 μM), 1 μl cDNA, and finally sterile double distilled water was added to the total volume. The qRT-PCR reaction procedure was at 95°C for 3 min, 39 cycles at 10 s and 58°C for 30 s. Melt curve was obtained after the last qRT-PCR cycle: 95°C for 5 s followed by a constant increase of 0.5°C in the temperature between 65 and 95°C. The changes in *TbMYC4A* expression in the seeds of Crocus and Z18-1244 at 14, 22, 28, and 35 dpa were examined. The relative quantitation method (2^–ΔΔ^CT) was used to calculate the fold changes in the expression levels of the target genes (Schefe et al., 2006).

## Results

### Transcriptome Analyses of Blue (Z18-1244) and White Aleurone (Crocus)

After filtering out low-quality reads, 27,664,008 and 23,288,416 clean reads were found in Crocus and Z18-1244, with GC contents of 53.93 and 56.35% and Q30 percentages of 93.56 and 93.91%, respectively. The alignment efficiency was 85.44% in Crocus and 87.17% in Z18-1244 according to sequence alignment against the reference genome. The transcripts per kilobase of fragment per million fragments were used to estimate quantitative gene expression levels. There were 9820 DEGs between Z18-1244 and Crocus according to the parameters of an FDR < 0.05 and a | log2 (fold change)| ≥ 1 (Supplementary Figure 1).

GO assignments classify annotated DEGs in terms of biological processes, molecular functions, and cellular components. Since elevated anthocyanin contents are generally caused by gene upregulation, upregulated genes were subjected to enrichment analysis. The subcomponents with *p*-values < 0.05 in each of the categories are depicted in bar graphs (Supplementary Figure 2). In the molecular function category, the greatest enrichment was observed for the activity of various enzymes and transporters, such as glutathione transferases, oxidoreductases, ATPase, and quercetin glycosyltransferase. In the biological process category, the annotated DEGs were enriched in secondary metabolite biosynthetic processes, flavonoid biosynthetic processes, flavonoid glucuronidation and other processes related to plant metabolites. In contrast, only one component was highly significantly enriched in the cellular component category.

**FIGURE 1:**
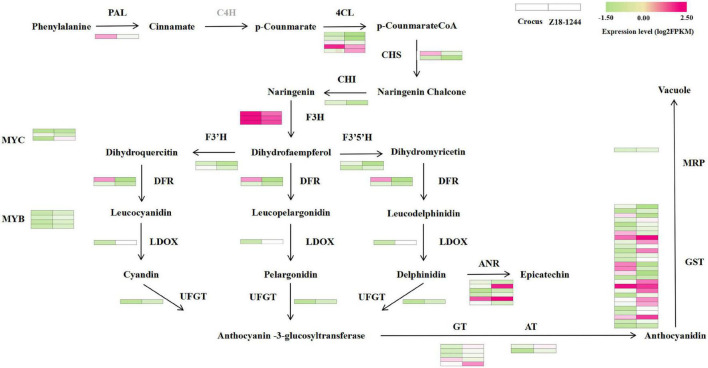
Expression profiles of DEGs [|log2(fold change)| ≥ 1, FDR < 0.05] related to the anthocyanin biosynthesis and transport processes in blue and white aleurone. The color scale from green (low) to purple (high) represents the log2FPKM values measured in white and blue aleurones.

The top 20 items in the KEGG enrichment results were selected for mapping in order of their *P*-values (Supplementary Figure 3). KEGG is mainly enriched in pathways associated with various nutrients, such as biosynthesis of amino acids, starch and sucrose metabolism, fructose and mannose metabolism. To find the key genes that control the blue aleurone layer, 12 structural genes and two transcription factors from the anthocyanin biosynthesis pathway were identified in the KEGG database to perform BLAXT analysis of the DEGs. Anthocyanin modification-related transferases and anthocyanin transport proteins were combined with the above structural genes to build a roadmap of anthocyanin biosynthesis and transport (Figure 1). A total of 41 unigenes were associated with anthocyanin biosynthesis and 29 to transport. No genes homologous to C4H were found. Among these genes, *LDOX*, *F3′5′H*, *UFGT*, *AT*, *GT*, *GST*, and *MRP* expression was elevated. Since the 4A^b^(4B) substitution line and *Ba2* from *T. boeoticum* were mapped to the centromeric region of 4AL, three genes located on chromosome 4 were screened out, including one structural gene (4CL, newGene_9199) and two transcription factor genes (one MYB, newGene_21086, and one bHLH (*TbMYC4A*), newGene_34829) (Supplementary Table 3). The MYB transcription factor of Z18-1244 was 100% homologous to that of Chinese spring, while the bHLH transcription factor was only 89.93% homologous. The expression level of this bHLH transcription factor (*TbMYC4A*) was 0 in Crocus, and the log2FoldChange between Crocus and Z18-1244 reached 6.14. Therefore, *TaMYC4A* was inferred as a candidate key gene for determining the blue aleurone layer trait in the 4A^b^(4B) substitution line Z18-1244 and *T. boeoticum.*

### Molecular Characteristics of *TbMYC4A*

**FIGURE 2:**
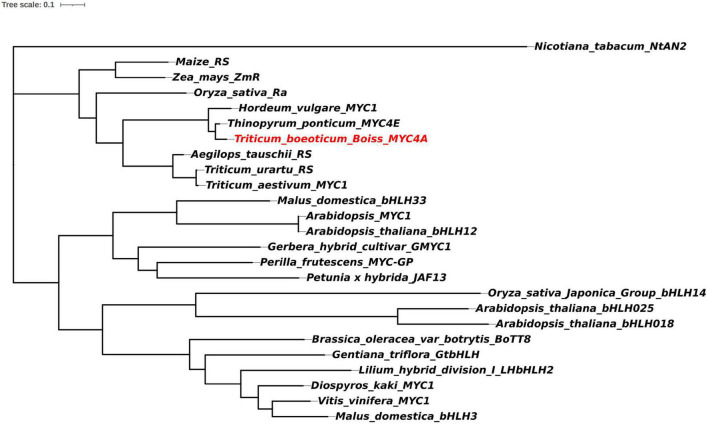
Phylogenetic tree of bHLH proteins regulating anthocyanin biosynthesis. The accession numbers of these proteins (or translated products) in the GenBank database are as follows: *Nicotiana tabacum*/NtAN2:ACO52472.1; *Oryza*/OsbHLH14:XP_015649359.1; *Arabidopsis thaliana*/bHLH025:Q9T072.2; *Arabidopsis thaliana*/bHLH018:Q1PF17.1; *Brassica oleracea var.botrytis*/BoTT8: ADP76654.1; Gentiana triflora/GTbHLH1:BAH03387.1; *Lilium hybrid division I/*LHbHLH2:BAE20058.1; *Diospyros kaki*/MYC1:AEC03343.1; *Vitis vinifera/*MYC1: ACC68685.1; *Malus domestica*/MdbHLH3:ADL36597.1; *Malus domestica*/MdbHLH33:AAC49219.1; *Arabidopsis thaliana*/MYC1:NP_191957; *Arabidopsis thaliana*/bHLH12:AAL55719.1; *Gerbera hybrid cultivar*/GMYC1: CAA07615.1; *Perilla frutescens*/MYC-GP:BAA75514.1; *Petunia x hybrida*/JAF13: AAC39455.1; *Oryza*/Ra:AAC49219; *Maize*/RS:NP_001106073; *Zea mays*/ZmR: NP_001105339.2; *Hordeum_vulgare*/MYC2:MF679157.1; *Thinopyrum ponticum/*MYC4E:KX914905.1; *Aegilops tauschii*/R-S:KD566857.1; *Triticum urartu*/RS: KD032825.1; and *Triticum aestivum*/MYC1:KY499900.1.

**FIGURE 3:**
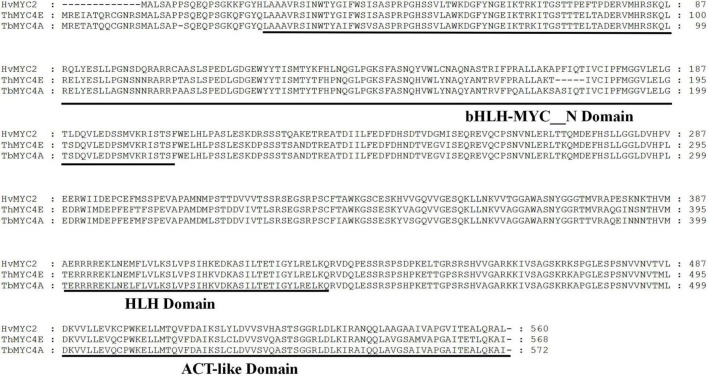
Comparison of the amino acid sequence of *TbMYC4A* with those of *ThNYC4E*, *HvMYC2*, and bHLH transcription factors known to regulate anthocyanin biosynthesis from *Thinopyrum ponticum* and *Hordeum vulgare*, with black lines representing bHLH-MYC_N, HLH and ACT-like domains. The accession numbers of these proteins (or translated product) in the GenBank database are as follows: *Thinopyrum ponticum*/MYC4E:KX914905.1 and *Hordeum vulgare*/MYC2:MF679157.1.

To further understand the evolution of this gene, a phylogenetic tree was constructed using the full-length amino acid sequences of bHLH transcription factors associated with anthocyanin biosynthesis via the adjacency matrix method with a bootstrap test of phylogeny performed with 1,000 replicates (Figure 2). *TbMYC4A*, encoding a protein of 572 amino acids, belonged to a branch including the bHLH proteins regulating anthocyanin biosynthesis in *Th. ponticum* and *H. vulgare*. *TbMYC4A* did not cluster with sequences of *T. urartu*, *Ae. tauschii*, and *T. aestivum.* In the phylogenetic tree, *TbMYC4A* was most closely related to *ThMYC4E* from *Th. ponticum* and *HvMYC2* from *H. vulgare*, which have been shown to be the key genes controlling the blue aleurone trait in the corresponding species. *TbMYC4A* also contained three complete functional domains, bHLH-MYC_N, HLH and ACT-like (Figure 3), which are essential for bHLH proteins to exert their transcriptional function.

**FIGURE 4:**
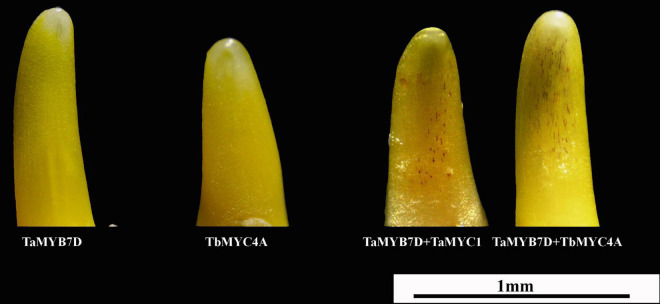
Wheat coleoptiles 16 h after bombardment with different plasmids. *TaMYB7D*, *TbMYC4A*, and *TaMYC1* represent the constructed vectors pBRACT214:TaMYB7D, pLGY-02: TbMYC4A, and pBRACT214:TaMYC1, respectively.

A bHLH transcription factor was shown to induce anthocyanin biosynthesis when coexpressed with the MYB transcription factor *TaMYB7D* (Zong et al., 2017). In this study, *TaMYC1* and *TbMYC4A* induced anthocyanin biosynthesis in the presence of *TaMYB7D* in “Opata” coleoptile cells; however, *TaMYC1*, *TbMYC4A* or *TaMYB7D* could not induce anthocyanin biosynthesis alone (Figure 4). The average number of cells accumulating anthocyanins was 35 or 50 in the coleoptiles simultaneously transfected with the *TaMYC1* and *TaMYB7D* or *TbMYC4A* and *TaMYB7D* expression vectors, respectively. Therefore, *TbMYC4A* should have a similar function to *TaMYC1* in regulating anthocyanin biosynthesis.

### Specific Marker Development for *TbMYC4A* and the Distribution of *TbMYC4A* in Natural Populations

**FIGURE 5:**
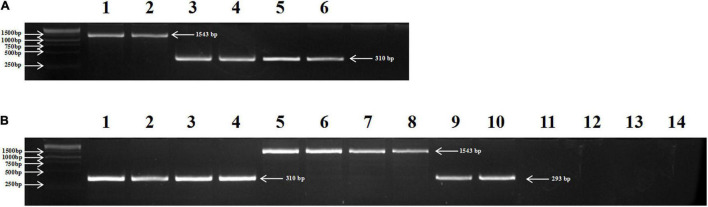
Distribution of *TbMYC4A* in Z18-1244, Crocus, G52, and 14 examples selected from 7 Triticeae species. **(A)** lanes 1 and 2: Crocus; lanes 3 and 4: *T. boeoticum* G52; lanes 5 and 6: Z18-1244; **(B)** *T. monococcum* accessions PI 10474T (1) and PI 168805 (2); *T. boeoticum* accessions PI 401416 (3) and PI 427506 (4); *T. timopheevii* accessions AS270 (5) and AS272 (6), *T. zhukovskyi* accessions TRI 7270-1 (7) and TRI 7270-3 (8); *Ae. tauschii* accessions AS60 (9) and AS62 (10); *T. urartu* accessions PI428224 (11) and PI428274 (12); and *T. turgidum* accessions PI525355 (13) and AS2239 (14).

To distinguish *TbMYC4A* from homologous genes in *T. aestivum*, the TbMYC4Aa1-F and TbMYC4Aa1-R primers were designed based on the coding sequences. The primers amplified a product of 310 bp from the genomic DNA of the blue-grained lines Z18-1244 and its male parent, *T. boeoticum* G52, but a 1543 bp product was amplified in white-aleurone Crocus (Figure 5A). Sequencing showed that the 310 bp amplification product of Z18-1244 and G52 was the same as the coding sequence of *TbMYC4A*, with an intron of 154 bp. It was found that Crocus had 1414 bp intron in the first and second exons, Z18-1244 had 154 bp in length and *Ae. tauschii* had 250 bp. A total of 24 genotypes, including 2 *T. urartu*, 4 *Ae. tauschii*, 4 *turgidum*, 2 *T. araraticum*, 2 *T. zhukovskyi*, 4 *T. monococcum*, and 6 *T. boeoticum* genotypes, were checked using the specific primers (Supplementary Table 1). Among the tested genotypes, the *TbMYC4A* sequence was also detected in other *T. boeoticum* accessions and its corresponding cultivated species, *T. monococcum*, which has the blue aleurone trait (Figure 5B). A product of slightly less length than *TbMYC4A* was detected in *Ae. tauschii*, which was sequenced to be 293 bp in length and contained a 250 bp intron. Since the homolog of *TbMYC4A* in wheat is localized on the D genome, no product were amplified in *T. urartu* (A^u^A^u^), and *T. turgidum* (AABB), but fragments of the same size as Crocus were amplified in *T. araraticum* (A^t^A^t^GG), and *T. zhukovskyi* (AAA^t^A^t^GG), which contains the G genome (Figure 5B).

### Expression Profile Analysis of *TbMYC4A*

**FIGURE 6:**
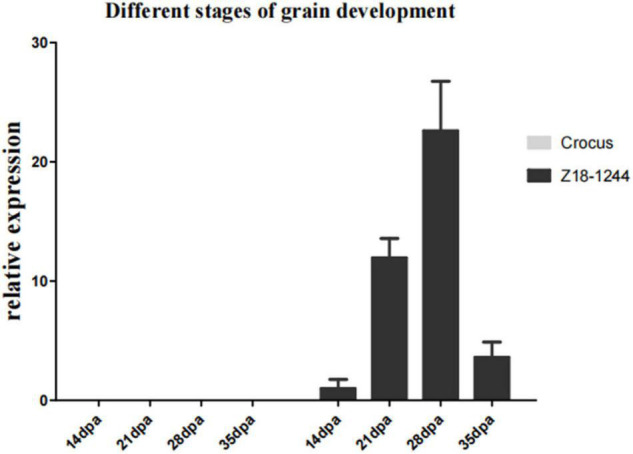
Relative expression of *TbMYC4A* in the seeds of Z18-1244 and Crocus at different developmental stages.

The expression of *TbMYC4A* in different developmental stages of seeds was observed by quantitative real-time PCR using immature seeds of Z18-1244 and Crocus at 14, 22, 28, and 35 dpa. The expression of *TbMYC4A* gradually increased at 14 dpa, 22 dpa, and 28 dpa and decreased considerably at 35 dpa in Z18-1244 (Figure 6). The expression level was 0 in all periods in Crocus, indicating that it does not express *TbMYC4A*, consistent with the transcriptomic results (Figure 6). The seeds of Crocus showed no color appearance in different developmental periods, while Z18-1244 showed a blue color visible to the naked eye at 22 dpa. Then the color deepened gradually, and the blue paste powder layer covered the whole seeds at 28 dpa. Then, the color did not change significantly, which was consistent with the qPCR results (Supplementary Figure 4).

## Discussion

Cyanidin-derived anthocyanins give some rice and maize varieties a red color, while no blue-grained trait has ever been found in rice or maize (Abdel-Aal et al., 2006; Sharma et al., 2011). However, the blue aleurone trait is present in several species of Triticeae, such as *Th. ponticum*, *T. boeoticum*, *H. vulgare*, and *Secale* species (Zeller et al., 1991; Li et al., 2017; Zong et al., 2019). Through transcriptome analysis, 77 genes related to anthocyanin biosynthesis were screened in an 4E additional line of common wheat-*Th. ponticum*, of which only eight genes were up-regulated in expression in blue paste-powder layer material, including five bHLH transcription factors and similarly. In addition, 78 genes were identified in blue-grained triticale, most of which were upregulated over the white aleurone layer and also included a MYB and a MYC transcription factor (*TsMYC2*, a candidate gene for control of wheat blue grain) (Li et al., 2017; Zong et al., 2019). In this study, RNA-Seq was used to identify the gene network controlling the blue aleurone trait of Z18-1244 wheat derived from *T. boeoticum*. In total, 41 unigenes were found to be associated with anthocyanin biosynthesis in Z18-1244. Similar to previous studies, the upregulated genes mainly included *LDOX*, *F3′5′H*, *UFGT*, *AT*, *GT*, most of which are late biosynthetic genes. *LDOX*, *F3′5′H* and *UFGT* are necessary for the synthesis of anthocyanins. Anthocyanins need to be transported to the vesicle for their bright color to be observed, and the most complete version of this process is probably carried out by a combination of *GST* located in the cytoplasm and *MRP* located in the vesicle membrane (Winkel-Shirley, 2001; Gu et al., 2019). Increases in anthocyanin contents are inextricably linked to these genes. To date, there have been few studies on the anthocyanin transport related genes. This study identified 29 anthocyanin transport related genes, most of which were up-regulated in blue aleurone layer materials relative to white aleurone layer materials.

bHLH transcription factor genes localized on chromosome 4 are considered the most likely candidates for various blue aleurone genes (Finch and Simpson, 1978; Zeven, 1991; Finch and Zong et al., 2019). This study identified the bHLH transcription factor *TbMYC4A* from the 4A^b^(4B) substitution line. It had the characteristic bHLH-MYC_N, HLH and ACT-like domains. In the evolutionary tree composed of bHLH proteins regulating anthocyanin biosynthesis, *TbMYC4A* was most closely related to the blue grain candidate genes *ThMYC4E* in *Th. ponticum* and *HvMYC2* in *H. vulgare*. The transient expression of *TbMYC4A* from *T. boeoticum* confirmed that it was a functional bHLH transcription factor with the ability to regulate anthocyanin biosynthesis. The *TbMYC4A*-specific marker was present in blue-grained common wheat Z18-1244, *T. boeoticum* and *T. monococcum* but absent in *T. urartu*, *Ae. tauschii*, *T. turgidum*, *T. araraticum*, and *T. zhukovskyi.* This indicates that the blue aleurone genes of *T. boeoticum* and its cultivated species, *T. monococcum*, are the same, consistent with previous studies (Zeller et al., 1991; Singh et al., 2007; Yu et al., 2017). qPCR experiments showed that the expression of *TbMYC4A* was rapidly elevated from 14 dpa to 28 dpa and decreased considerably at 35 dpa in Z18-1244, consistent with the anthocyanin accumulation pattern. *TbMYC4A* was not expressed in any stage of seed development in non-blue-grained Crocus wheat. Several studies have shown that amino acids, trace elements and other nutrients are increased in blue-grained wheat relative to common wheat, as is the anthocyanin content (Tian et al., 2018). In this study, GO and KEGG enrichment analyses of Z18-1244 and Crocus revealed that upregulated differentially expressed genes were enriched in various nutrient-related pathways, including flavonoid biosynthesis, amino acid biosynthesis, zinc and iron transport-related pathways, starch and sucrose metabolism, and nitrogen metabolism (Supplementary Figures 1, 2). It has been indicated that blue aleurone presents some advantages over white aleurone in terms of the accumulation of certain nutrients in addition to anthocyanins. This result implies that the anthocyanin pathway may affect other nutrient-related biosynthesis pathways.

## Conclusion

In conclusion, a functional bHLH transcription factor, *TbMYC4A*, was isolated from the wheat-*T. boeoticum* substitution line Z18-1244, with the blue aleurone trait derived from *T. boeoticum*. All natural accessions of *T. boeoticum* and its cultivated species, *T. monococcum*, with the blue aleurone trait carried this gene. It was indicated that *TbMYC4A* was the candidate *Ba2* gene that controlled the related trait. No blue-grained varieties containing the *Ba2* gene have been released to date. These results will help to explore the molecular mechanism of blue aleurone formation in *T. boeoticum* and will help to breed wheat varieties with blue aleurone.

## Data Availability Statement

The datasets presented in this study can be found in online repositories. The names of the repository/repositories and accession number(s) can be found below: https://www.ncbi.nlm.nih.gov/genbank/, MZ686958.

## Author Contributions

XL: formal analysis, investigation, data curation, writing—original draft, and visualization. MZ: formal analysis and investigation. XJ, HL, and ZJ: investigation. MH, BJ, LH, SN, ZY, XJC, and XC: supervision. DL: supervision, writing—review, and editing. BL: writing—review, and editing, supervision, and methodology. LZ: conceptualization, methodology, writing—review, editing, and project administration. All authors contributed to the article and approved the submitted version.

## Conflict of Interest

The authors declare that the research was conducted in the absence of any commercial or financial relationships that could be construed as a potential conflict of interest.

## Publisher’s Note

All claims expressed in this article are solely those of the authors and do not necessarily represent those of their affiliated organizations, or those of the publisher, the editors and the reviewers. Any product that may be evaluated in this article, or claim that may be made by its manufacturer, is not guaranteed or endorsed by the publisher.

## Footnotes

1
ftp://ftp.ncbi.nih.gov/blast/db/

2
https://www.UniProt.org/

3
http://www.ncbi.nlm.nih.gov/COG/

4
https://www.genome.jp/kegg/

5
http://www.geneontology.org/

6
ftp://ftp.ncbi.nih.gov/pub/COG/KOG/

7
http://pfam.xfam.org/

8
http://eggnog5.embl.de/

9
http://www.ncbi.nlm.nih.gov/genbank/

## Abbreviations

PAL: Phenylalanine lyase
4CL: 4-coumarate–CoA ligase
CHS: Chalcone synthase
CHI: Chalcone isomerase
F3H: Flavanone 3-hydroxylase
F3′H: Flavonoid 3′-hydroxylase
F3′5′H: Flavonoid 3′5′-hydroxylase
DFR: Dihydroflavonol 4-reductase
LDOX: Leucoanthocyanidin dioxygenase
UFGT: Anthocyanidin3-O-glucosyltransferase
MYB: Myeloblastosis transcription factor
MYC: Basic helix-loop-helix transcription factor
ANR: Anthocyanidin reductase
GT: Glucosyltransferase
AT: Acyltransferase
GST: Glutathione transferase
MRP: Multidrug-resistance associated protein
dpa: Days post-anthesis.
