# Supplementary material for: TbMYC4A Is a Candidate Gene Controlling the Blue Aleurone Trait in a Wheat-Triticum boeoticum Substitution Line
Source: Front Plant Sci. 2021 Nov 5;12:762265. doi: 10.3389/fpls.2021.762265 (PMC8603940; doi:10.3389/fpls.2021.762265)
Supplement: Supplementary file 1 [file Table_1.DOCX]

**Table.S1 The phenotype and genotype of materials used to detect the presence or lack of *TbMYC4A* in this study.**

| Item | Taxon | Plant ID | Phenotype | *TbMYC4A* |
| --- | --- | --- | --- | --- |
| 1 | *T. turgidum* ssp*. turanicum* | PI532136 | White | - |
| 2 | *T. turgidum* ssp*. turanicum* | PI525355 | White | - |
| 3 | *T. turgidum* ssp*. turgidum* | AS2239 | White | - |
| 4 | *T. turgidum* ssp*. turgidum* | AS2240 | White | - |
| 5 | *Ae. tauschii* | AS60 | White | - |
| 6 | *Ae. tauschii* | AS62 | White | - |
| 7 | *Ae. tauschii* | AS63 | White | - |
| 8 | *Ae. tauschii* | AS65 | White | - |
| 9 | *T. urartu* | PI428224 | White | - |
| 10 | *T. urartu* | PI428274 | White | - |
| 11 | *T. araraticum* | AS270 | White | - |
| 12 | *T. araraticum* | AS272 | White | - |
| 13 | *T. zhukovskyi* | TRI 7270-1 | White | - |
| 14 | *T. zhukovskyi* | TRI 7270-3 | White | - |
| 15 | *T. monococcum* | PI 10474T | Blue | + |
| 16 | *T. monococcum* | PI 168805 | Blue | + |
| 17 | *T. monococcum* | PI 266844 | Blue | + |
| 18 | *T. monococcum* | PI 272535 | Blue | + |
| 19 | *T. boeoticum* | PI 401416 | Blue | + |
| 20 | *T. boeoticum* | PI 427506 | Blue | + |
| 21 | *T. boeoticum* | PI 427510 | Blue | + |
| 22 | *T. boeoticum* | PI 427514 | Blue | + |
| 23 | *T. boeoticum* | PI 427662 | Blue | + |
| 24 | *T. boeoticum* | PI 427749 | Blue | + |

AS codes refer to the materials provided by Triticeae Research Institute, Sichuan Agricultural University. PI codes refer to the materials from USDA-ARS, NSGC (https://npgsweb.ars grin.gov/gringlobal/search.aspx?). TRI refer to the materials from USSR. ‘+’ represent the presence of *TbMYC4A* in the fourth column and ‘-’ represent the missing of *TbMYC4A*.
