# Supplementary material for: TbMYC4A Is a Candidate Gene Controlling the Blue Aleurone Trait in a Wheat-Triticum boeoticum Substitution Line
Source: Front Plant Sci. 2021 Nov 5;12:762265. doi: 10.3389/fpls.2021.762265 (PMC8603940; doi:10.3389/fpls.2021.762265)
Supplement: Supplementary file 2 [file Table_2.DOCX]

**Table.S2** **Names and sequences of the primers used in this study.**

| Number | Primer | Sequence (5'-3') |
| --- | --- | --- |
| 1 | TbMYC4Acds-F | ATGCGGGAAACAGCTACTCAG |
| 2 | TbMYC4Acds-R | CTATATAGCTTTCTGAAGCGCTTCA |
| 3 | TbMYC4AF2 | TCTAGAGGATCCCCGATGCGGGAAACAGCTACTCAGCAGT |
| 4 | TbMYC4AR2 | TTCGAGCTCTCTAGACTATATAGCTTTCTGAAGCGCTTCA |
| 5 | GAPDH-F | TGTCCATGCCATGACTGCAA |
| 6 | GAPDH-R | CCAAGTGCTTGGAATGATG |
| 7 | TbMYC4A-RT-F | ACCCTAATCAAGGGTTGCCAG |
| 8 | TbMYC4A-RT-R | CAAACGATTGTCTGAATAGAGGC |
| 9 | TbMYC4Aa1-F | CGCTATCAGCTCCCAGTCAG |
| 10 | TbMYC4Aa1-R | CATCCTTCCACGCCAGAACT |
