# Supplementary material for: TbMYC4A Is a Candidate Gene Controlling the Blue Aleurone Trait in a Wheat-Triticum boeoticum Substitution Line
Source: Front Plant Sci. 2021 Nov 5;12:762265. doi: 10.3389/fpls.2021.762265 (PMC8603940; doi:10.3389/fpls.2021.762265)
Supplement: Supplementary file 3 [file Table_3.DOCX]

**Table.S3 The information of unigenes relative to anthocyanin biosynthesis and [transport](E:/%E6%9C%89%E9%81%93/Dict/8.9.6.0/resultui/html/index.html#/javascript:;) in aleurones.**

| **Gene** | **GeneID** | **Chromosome**  **location** | **White-Expression** | **Blue-Expression** | **DFR** | **Log2FoldChange**  **(red/blue)** | **Up/down**  **(red/blue)** |
| --- | --- | --- | --- | --- | --- | --- | --- |
| PAL | newGene_161687 | Chr2A | 9.26 | 3.35 | 0.002417852 | -1.448970071 | down |
| 4CL | newGene_22961 | Chr4B | 0.63 | 0.00 | 8.75E-10 | -4.876450917 | down |
|  | newGene_28918 | Chr4B | 1.20 | 0.00 | 1.22E-14 | -5.716511043 | down |
|  | newGene_82268 | Chr6A | 5.25 | 2.43 | 1.02E-05 | -1.229322883 | down |
|  | newGene_89468 | Chr6B | 25.44 | 9.61 | 0.012574783 | -1.401898369 | down |
|  | newGene_9199 | Chr4A | 2.00 | 9.91 | 3.56E-16 | 2.059710492 | up |
| CHS | TraesCS2A02G537400 | Chr2A | 8.21 | 2.03 | 2.29E-05 | -2.002337693 | down |
|  | newGene_151168 | Chr2A | 0.69 | 0.03 | 6.91E-05 | -3.164892762 | down |
| CHI | newGene_154898 | Chr2A | 1.45 | 0.36 | 0.000983737 | -1.947663277 | down |
| F3H | newGene_102569 | Chr6D | 2.24 | 6.96 | 0.011405009 | 1.271029072 | up |
|  | newGene_164208 | Chr2A | 39.56 | 13.95 | 5.86E-05 | -1.637510217 | down |
|  | newGene_177213 | Chr2B | 55.74 | 17.08 | 1.03E-06 | -1.842069785 | down |
|  | newGene_188543 | Chr2D | 39.19 | 17.99 | 0.001633965 | -1.296811873 | down |
| F3'H | newGene_150600 | Chr1D | 1.00 | 0.28 | 0.027943849 | -1.69240107 | down |
|  | newGene_24143 | Chr1A | 3.68 | 1.60 | 0.0068527 | -1.312654139 | down |
| F3'5'H | TraesCS4B02G151700 | Chr4B | 2.11 | 0.06 | 2.09E-10 | -4.163833285 | down |
|  | newGene_103266 | Chr6D | 0.49 | 1.77 | 0.003923722 | 1.507089265 | up |
| DFR | newGene_114965 | Chr7A | 10.32 | 0.00 | 7.39E-35 | -7.843028544 | down |
|  | newGene_3031 | Chr3D | 1.67 | 0.65 | 0.000418436 | -1.597214747 | down |
| LDOX | newGene_96252 | Chr6D | 0.45 | 4.50 | 5.19E-10 | 2.906185372 | up |
| UFGT | newGene_94888 | Chr6B | 0.06 | 1.22 | 0.00031834 | 2.877695944 | up |
| MYB | newGene_104685 | Chr7A | 0.25 | 1.59 | 0.00965875 | 1.911726434 | up |
|  | newGene_129932 | Chr7D | 0.84 | 2.75 | 0.045977856 | 1.276948944 | up |
|  | newGene_21086 | Chr4A | 0.15 | 1.79 | 3.70E-07 | 2.944042534 | up |
|  | newGene_59381 | Chr5B | 0.36 | 1.52 | 0.002892517 | 1.717253467 | up |
| MYC | newGene_172542 | Chr2B | 0.05 | 0.36 | 0.006056209 | 1.991270356 | up |
|  | newGene_28055 | Chr4B | 2.68 | 1.19 | 0.009438781 | -1.204301982 | down |
|  | newGene_34829 | Chr4D | 0.00 | 5.22 | 1.65E-19 | 6.140605145 | up |
| ANR | newGene_188327 | Chr2D | 2.06 | 1.17 | 9.90E-06 | -2.357902733 | down |
|  | newGene_188329 | Chr2D | 2.86 | 24.58 | 3.11E-08 | 2.724794001 | up |
|  | newGene_163931 | Chr2A | 0.00 | 0.84 | 1.50E-07 | 4.211306731 | up |
|  | newGene_177025 | Chr2B | 0.63 | 2.47 | 0.001622218 | 1.59840902 | up |
|  | newGene_177020 | Chr2B | 16.74 | 52.26 | 1.33E-06 | 1.393668589 | up |
|  | newGene_163930 | Chr2A | 3.36 | 1.42 | 0.005830211 | -1.263238923 | down |
| GT | newGene_49317 | Chr5A | 1.28 | 5.09 | 0.000279164 | 1.680779285 | up |
|  | newGene_49320 | Chr5A | 0.89 | 3.51 | 1.05E-05 | 1.6655696 | up |
|  | newGene_104732 | Chr1B | 0.93 | 3.55 | 1.42E-06 | 1.665816158 | up |
|  | newGene_199097 | Chr3A | 5.83 | 2.65 | 0.002923178 | -1.267787355 | down |
|  | newGene_211522 | Chr3B | 3.73 | 10.77 | 0.015365427 | 1.185957999 | up |
| AT | newGene_30064 | Chr4D | 2.03 | 5.02 | 0.02145994 | 1.010346649 | up |
|  | newGene_189112 | Chr2D | 0.04 | 2.59 | 4.67E-15 | 4.79681209 | up |
| GST | newGene_29008 | Chr4B | 2.40 | 0.00 | 6.97E-15 | -5.739182157 | down |
|  | newGene_127629 | Chr7B | 0.00 | 1.59 | 2.10E-17 | 5.86618115 | up |
|  | newGene_22359 | Chr4B | 5.78 | 0.16 | 1.60E-14 | -4.574230615 | down |
|  | newGene_207694 | Chr3B | 1.83 | 5.05 | 0.002744301 | 1.207947963 | up |
|  | newGene_215691 | Chr3B | 0.00 | 2.86 | 2.85E-12 | 5.1698635 | up |
|  | newGene_72585 | Chr1B | 0.70 | 2.35 | 0.019242697 | 1.428562894 | up |
|  | newGene_96388 | Chr6D | 8.09 | 1.83 | 3.14E-07 | -2.152118677 | down |
|  | newGene_17256 | Chr4A | 14.94 | 38.74 | 4.39E-07 | 1.170280596 | up |
|  | newGene_586 | Chr3D | 2.10 | 10.22 | 1.51E-09 | 2.007006986 | up |
|  | newGene_211156 | Chr3B | 1.15 | 4.03 | 0.018525389 | 1.485861049 | up |
|  | newGene_10689 | Chr4A | 0.17 | 12.07 | 1.20E-85 | 5.734215437 | up |
|  | TraesCS4D02G044400 | Chr4D | 1.07 | 2.77 | 0.000597338 | 1.122890505 | up |
|  | newGene_146096 | Chr1D | 0.77 | 4.55 | 2.45E-10 | 2.240774305 | up |
|  | newGene_26619 | Chr4B | 12.26 | 0.15 | 0.000132192 | -3.471439901 | down |
|  | newGene_26618 | Chr4B | 12.65 | 1.18 | 6.80E-12 | -3.264743093 | down |
|  | newGene_22361 | Chr4B | 5.82 | 0.00 | 4.05E-23 | -6.742725255 | down |
|  | newGene_179891 | Chr1A | 0.08 | 0.77 | 0.047885786 | 1.920906959 | up |
|  | newGene_4362 | Chr3D | 3.19 | 13.34 | 0.000149947 | 1.786744235 | up |
|  | newGene_45971 | Chr1B | 37.08 | 21.06 | 8.04E-06 | -1.015218611 | down |
|  | newGene_207697 | Chr3B | 3.09 | 14.08 | 2.32E-10 | 1.881204492 | up |
|  | newGene_61214 | Chr5B | 0.05 | 3.02 | 5.17E-14 | 4.517341991 | up |
|  | newGene_49441 | Chr1B | 4.39 | 10.75 | 6.41E-05 | 1.060322895 | up |
|  | newGene_188730 | Chr2D | 3.36 | 9.04 | 0.00012706 | 1.176367341 | up |
|  | newGene_7371 | Chr3D | 0.20 | 3.92 | 6.95E-07 | 3.360307924 | up |
|  | TraesCS1A02G188000 | Chr1A | 0.86 | 4.33 | 8.28E-05 | 1.98952883 | up |
|  | newGene_10688 | Chr4A | 7.12 | 19.81 | 1.55E-05 | 1.264920757 | up |
|  | newGene_42571 | Chr5A | 0.67 | 3.23 | 9.52E-06 | 1.939099686 | up |
|  | newGene_45190 | Chr5A | 0.12 | 0.74 | 0.000448732 | 2.10705427 | up |
| MRP | newGene_5254 | Chr3D | 0.99 | 2.34 | 0.000929379 | 1.019641041 | up |
