# Supplementary material for: TbMYC4A Is a Candidate Gene Controlling the Blue Aleurone Trait in a Wheat-Triticum boeoticum Substitution Line
Source: Front Plant Sci. 2021 Nov 5;12:762265. doi: 10.3389/fpls.2021.762265 (PMC8603940; doi:10.3389/fpls.2021.762265)
Supplement: Supplementary file 4 [file Image_1.PDF]

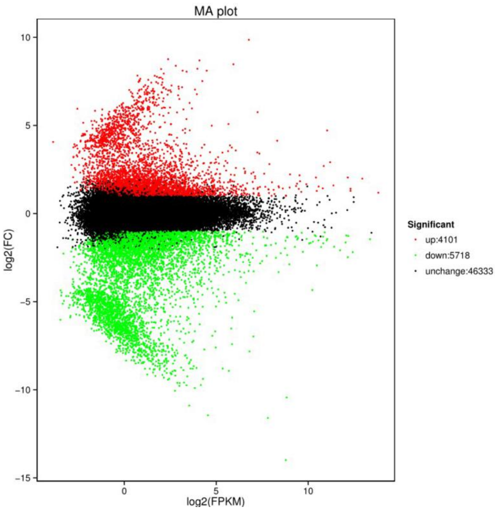

Fig.S1 Differentially expressed genes between Crocus and Z18-1244. The red gene is up-regulated if gene expression of Z18-1244 is higher than that of Crocus; the green gene is down-regulated if the gene expression of Crocus is higher than that of Z18-1244; and the black gene expression level shows no direct difference between the two.
