# Supplementary material for: TbMYC4A Is a Candidate Gene Controlling the Blue Aleurone Trait in a Wheat-Triticum boeoticum Substitution Line
Source: Front Plant Sci. 2021 Nov 5;12:762265. doi: 10.3389/fpls.2021.762265 (PMC8603940; doi:10.3389/fpls.2021.762265)
Supplement: Supplementary file 6 [file Image_3.PDF]

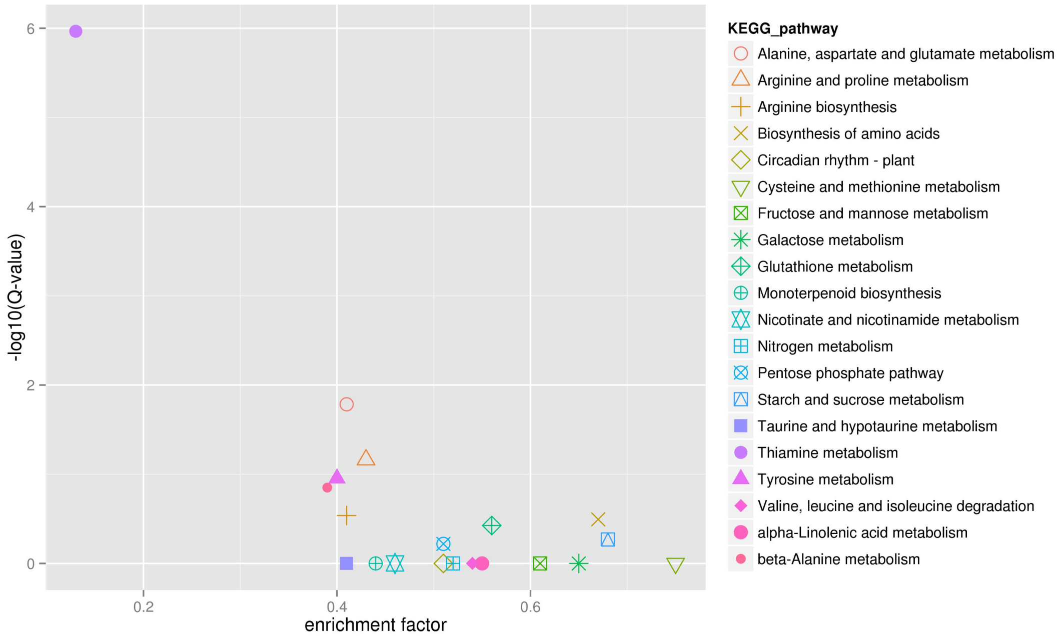

Fig.S3 Pathway enrichment analysis of up-regulated differentially expressed genes. The top 20 pathways with the lowest significant p-value were used for mapping.
