# Supplementary material for: TbMYC4A Is a Candidate Gene Controlling the Blue Aleurone Trait in a Wheat-Triticum boeoticum Substitution Line
Source: Front Plant Sci. 2021 Nov 5;12:762265. doi: 10.3389/fpls.2021.762265 (PMC8603940; doi:10.3389/fpls.2021.762265)
Supplement: Supplementary file 7 [file Image_4.PDF]

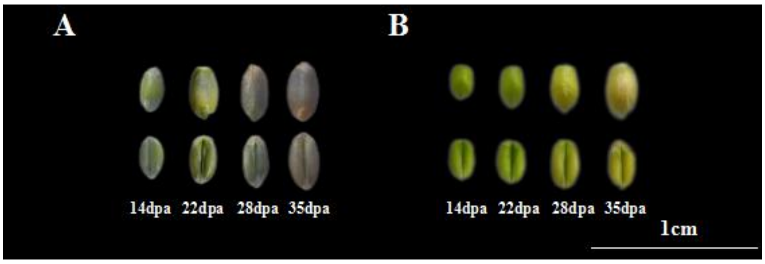

Fig.S4 The seed morphology of blue-grained substitution line Z18-1244 (A) and Crocus (B) at different stages of development.
